# Participation of the *arcR*_ACME_ protein in self-activation of the *arc* operon located in the arginine catabolism mobile element in pandemic clone USA300

**DOI:** 10.1590/0074-02760160424

**Published:** 2017-07

**Authors:** Zayda Lorena Corredor Rozo, Ricaurte Alejandro Márquez-Ortiz, Betsy Esperanza Castro, Natasha Vanegas Gómez, Javier Escobar-Pérez

**Affiliations:** 1 Universidad El Bosque, Bacterial Molecular Genetics Laboratory, Bogotá, Colombia; 2 University of Technology, Faculty of Science, I3 Institute, Infection, Immunity and Innovation, Sydney, Australia

**Keywords:** USA300 clone, ACME, transcriptional activator

## Abstract

*Staphylococcus aureus* pandemic clone USA300 has, in addition to its constitutive arginine catabolism (*arc*) gene cluster, an arginine catabolism mobile element (ACME) carrying another such cluster, which gives this clone advantages in colonisation and infection. Gene *arcR*, which encodes an oxygen-sensitive transcriptional regulator, is inside ACME and downstream of the constitutive *arc* gene cluster, and this situation may have an impact on its activation. Different relative expression behaviours are proven here for *arcR*_*ACME*_ and the *arc*_ACME_ operon compared to the constitutive ones. We also show that the artificially expressed recombinant ArcR_ACME_ protein binds to the promoter region of the *arc*_*ACME*_ operon; this mechanism can be related to a positive feedback model, which may be responsible for increased anaerobic survival of the USA300 clone during infection-related processes.

*Staphylococcus aureus* USA300 clone is characterised by its resistance to most β-lactam antibiotics, virulence, global spread, and association with invasive diseases (Diep et al. 2006). Under stressful conditions such as oxygen depletion, USA300 ensures its survival by glucose fermentation or by means of nitrates as alternative electron acceptors. However, in the absence of glucose or nitrates in the medium, arginine becomes an important alternative energy source. *S. aureus* has a constitutive arginine deaminase (ADI) pathway encoded by the arginine catabolism operon (*arc*_cons_) (Makhlin et al. 2007). This pathway has been identified in some facultative or absolute anaerobic eukaryotes (*Saccharomyces cerevisiae* and *Giardia intestinalis*) and has also broadly spread into prokaryotes, mainly facultative anaerobes such as *Streptococcus* spp., *Bacillus* spp., and *Staphylococcus* spp*.* among others (Barcelona-Andres et al. 2002, Zúñiga et al. 2002, Makhlin et al. 2007, Lindgren et al. 2014).

Sequencing of the USA300 clone genome led to identification of an alternative *arc* operon, which is transported in the arginine catabolism mobile element (ACME) (Diep et al. 2006). Although the *arc* operon in ACME (*arc*_ACME_) and constitutive *arc* operon (*arc*_cons_) have the same structural genes (*arcABDC*), they have different genetic arrangements (Fig. 1). These genes have sequence identity ranging from 56.7% to 75.5%, and their proteins have sequence identity and similarity ranging from 40.1% to 81.3% and 63.1% to 89.9%, respectively (Urushibara et al. 2012, Thurlow et al. 2013). In addition, the *arc*_ACME_ operon has a hypothetical open reading frame (ORF) of 690 bp inserted into its central region coding for a putative ArcR protein, as opposed to *arcR* in the constitutive operon; that is located downstream and has an independent promoter region (Diep et al. 2006) (Fig. 1). This ORF encodes a protein of 229 amino acid (aa) residues that belongs to the family of cAMP-CRP receptor proteins just as its homologous constitutive protein ArcR (234 aa) from *arc*_cons_ (Tonon et al. 2001, Ibarra et al. 2013). The biological implications of this rearrangement have not been assessed. We hypothesised that the rearrangement of the *arcR* gene inside the *arc*_ACME_ operon is energetically favourable for its self-activation, contributing to the adaptive nature of pandemic clone USA300.

**Fig. 1:**
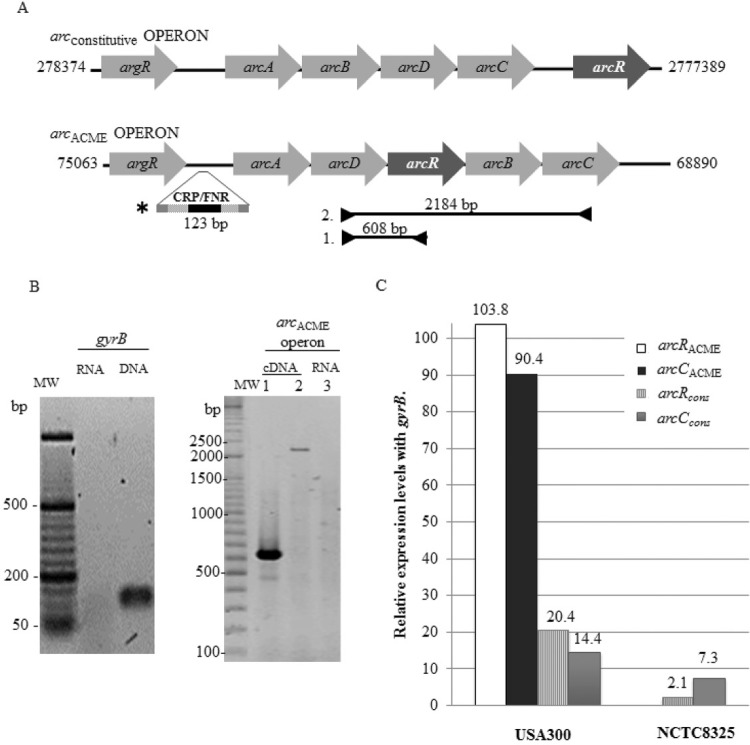
: differential expression of arginine catabolism (*arc*) operons and transcriptional regulators ArcR. (A) A diagram of *arc* operons in clone USA300. The inverted arrows indicate the location of primers used for evaluation of the co-transcription from arginine-ornithine antiporter gene (*arcD*) to carbamate kinase gene (*arcC*), and *arcD* to *arcR* in the arginine catabolism mobile element (*arc*ACME) operon. The fragment indicated by an asterisk (*) is the promoter probe that contains the Crp-binding motif TGTGA-N6-TCACA (dark box). (B) Co-transcription of *arcRACME* with the other genes of the *arc*ACME operon in strain USA300 under anaerobic conditions, as determined by polymerase chain reaction (PCR) using total cDNA as a template and primers 1 (lane 1) and 2 (lane 2) from panel A. Lane 3 is the same PCR analysis of RNA as a control to rule out DNA remnants. (C) Relative transcription levels of genes *arcRACME*, *arcCACME*, *arcRcons*, and *arcCcons* genes during anaerobiosis in the stationary phase (20 h; results are expressed as the mean of two different experiments).

In order to assess the role of *arcR* in transcription of the *arc*_ACME_ operon, total RNA was extracted using the TRIzol method from anaerobic trypticase soy broth (TSB) cultures (using the GENbag microaer system, BioMérieux®, and Resazurin, Oxoid®, to verify the anaerobic environment during the experiment) supplemented (or not) with arginine (50 mM, pH 7.2) and incubated at 37ºC for 20 h. Prior to all the experiments, RNA was treated with DNase (Promega), and the presence of genomic DNA contamination was ruled out because no *gyrB* gene amplification was detected by polymerase chain reaction (PCR) with the RNA as template. Integrity of the (DNA-free) RNA was assessed by agarose gel electrophoresis. From these RNA samples, cDNAs were synthesised using reverse transcriptase (MMLV RT, 200 U/μL), a 1:4 ratio of random hexamers to RNA, 0.5 mM dNTPs, and 1X buffer [50 mM Tris-HCl pH 8.3, 75 mM KCl, 3 mM MgCl_2_, and 10 mM dithiothreitol (DTT)] in a final volume of 25 μL [adjusted with diethyl pyrocarbonate (DEPC)-treated water]. This reaction was allowed to proceed for 1 h at 37ºC. Quantification of relative expression of constitutive *arcR* and *arcC* genes (*arcC*_*cons*_ and *arcR*_*cons*_) and the genes from ACME (*arcR*_*ACME*_, *arcC*_*ACME*_) was determined by quantitative PCR (qPCR) to treated (50 mM arginine) and untreated (without arginine) *S. aureus* USA300_FPR3757 and NCTC8325 strains (as a control of basal expression of the constitutive genes) strains in TSB cultures (Supplementary data, Tables I-II and methods). Relative amounts were calculated according to the method proposed by Schefe et al. (2006), with normalisation to housekeeping gene *gyrB* (Fig. 1C) (Schefe et al. 2006). Additionally, to test whether the ArcR_ACME_ protein binds to the regulatory region of the *arc*_ACME_ operon, rArcR_ACME_ (recombinant ArcR_ACME_ protein) was produced and purified from *Escherichia coli* BL21 (DE3) cells following the manufacturer recommendations (Thermo Scientific) (Supplementary data, Table I and methods). Protein expression was confirmed by western blotting using the monoclonal anti-6x-His tag antibody (Abcam; Fig. 2). A 123-bp fragment of the *arc*_ACME_ operon promoter was synthesised by PCR and labelled using biotin-11-dUTP (biotinylated probe; Supplementary data, Table II). The possible binding of rArcR_ACME_ to this biotinylated DNA was evaluated by means of electrophoretic mobility shift assay (EMSA), and formation of the protein-probe complex was detected by chemiluminescence. Specificity of the binding was assessed using specific and non-specific unbiotinylated competitors. In addition, the biotinylated probe and recombinant protein were cross-linked by mixing them followed by irradiation with UV light for 10 min. This product was separated in duplicate by SDS-PAGE and transferred to polyvinylidene difluoride (PVDF) and nylon membranes for western blotting and chemiluminescence assays, respectively.

**Fig. 2:**
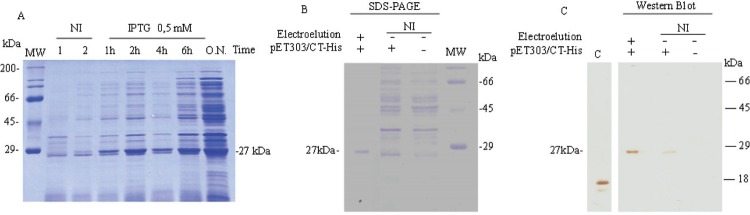
: expression, purification, and detection of the recombinant argenine transcriptional regulator arginine catabolism mobile element (rArcRACME) protein. (A) The amount of the rArcRACME protein in the insoluble fraction of a total protein extract from *Escherichia coli* BL21-arcR, induced with 0.5 mM isopropyl β-D-1-thiogalactopyranoside (IPTG) at 37ºC at different time points. Lane 1, *E. coli* BL21 (DE3) without recombinant plasmid pET303/CT-His. Lane 2, *E. coli* BL21 (DE3) with recombinant plasmid pET303/CT-His, not induced. rArcRACME (~27 kDa) was purified from overnight (O.N.) culture of *E. coli* BL21-arcR by electroelution and visualised by SDS-PAGE (B) and western blotting (C) using an anti-6x-His antibody. Lane C in the western blot is a positive control for the anti-His antibody. NI: not induced.

The ADI metabolic pathway is regulated by the ArgR and ArcR proteins acting as a repressor and an activator, respectively (Makhlin et al. 2007, Thurlow et al. 2013). The putative ArcR_ACME_ protein (GenBank accession number: WP_000272781.1), showed 40% sequence similarity with ArcR_cons_ from the constitutive operon (GenBank accession number: WP_000138214.1) in *S. aureus* and 99.9% identity with the orthologous protein identified in the *S. epidermidis* ATCC 12228 (GenBank accession number: NP_763659.1). Additionally, bioinformatics analysis of the ArcR_ACME_ protein identified a cAMP-binding domain from the CRP family (Conserved Domains Database accession number: COG0664) and a C-terminal helix-turn-helix DNA-binding motif (Conserved Domains Database accession number: cl21459), which are reported as defining characteristics of CRP/FNR transcription factors (Maghnouj et al. 2000, Körner et al. 2003, Gruening et al. 2006, Planet et al. 2013). To determine whether the *arcR*_*ACME*_ gene activation responds to the same stimuli as the *arc*_ACME_ operon does, we tried to find simultaneous co-transcripts from the remaining genes in the operon and *arcR*_*ACME*_ in the cDNA by means of PCR. Using the *arcD-arcC* primers and the *arcD-arcR*_*ACME*_ primers (Fig. 1A), we found that the *arcR*_*ACME*_ gene is co-transcribed with the other *arc*_ACME_ operon genes (Fig. 1A-B). This result suggests that when *arc*_ACME_ is activated, the ArcR_ACME_ protein is expressed along with the *arc*_ACME_ proteins. This means that the *arcR*_ACME_ gene responds to the same activating stimuli as the *arc*_ACME_ operon does, contrary to the activation of the *arc*_cons_ operon and *arcR*_*cons*_ gene, which respond to different stimuli, because the *arcR*_*cons*_ gene has an independent promoter region (Makhlin et al. 2007). In order to determinate the possible impact of this differential activation of the two *arc* operons, a qPCR analysis was carried out as mentioned above. It was found that under anaerobic conditions, the *arc*_ACME_ operon in strain USA300 was transcribed 90-fold more abundantly than the *arc*_cons_ operon was, as inferred from expression of the *arcC* gene (Fig. 1C). Additionally, the *arcR*_*ACME*_ gene was transcribed 83-fold more abundantly than the *arcR*_*cons*_ gene was (Fig. 1C). These results suggest that ArcR_ACME_ may have a positive feedback effect on the *arc*_ACME_ operon because minimal quantities of this transcriptional regulators can increase transcription of the whole operon and in turn, also increase its own production. Furthermore, it is possible that the increase in the transcription of *arcR*_ACME_ has an additional effect upon the constitutive *arc* operon because an increase in its transcription above its basal expression level is also observed in the NCTC8325 strain without ACME (a sevenfold smaller increase, Fig. 1C). However, the influence of additional unexplored factors could not be ruled out in the analysis of the differences in transcription observed between these two strains.

Alignments of the promoter sequences of the two *arc* operons in strain USA300 showed relatively low nucleotide identity (43.9%). However, transcription basic elements and a putative binding site for CRP-regulatory proteins (TTGTGA-N_6_-TCACA) were found to be conserved (Fig. 3C) (Makhlin et al. 2007, Ibarra et al. 2013, Matsui et al. 2013). EMSA experiments with a biotinylated 123-bp double-stranded DNA fragment corresponding to a part of the ACME promoter, encompassing the TTGTGA-N_6_-TCACA hypothetical ArcR-binding site, revealed gel retardation by rArcR_ACME_. Additionally, this rArcR_ACME_ electrophoretic shift was prevented by a specific competitor (the same probe without biotin; Figs 1A, 3A) but not by a non-specific competitor (91-bp *gyrB* fragment). These results confirmed the specific binding of rArcR_ACME_ to the promoter region of the *arc*_ACME_ operon. Furthermore, rArcR_ACME_ incubated with the promoter region probe (Fig. 3B) and later UV-cross-linked so that it covalently binds to the DNA interacting with the recombinant protein, showed a single signal in the western blot (corresponding to the recombinant protein) and three signals in the chemiluminescence assay, one of which co-localised with rArcR_ACME_, thus confirming formation of a protein-probe complex. However, when we used a total protein extract, it was impossible to detect formation of this protein-probe complex, possibly because of a low concentration of the native protein in this total extract or because in the total extract, the native protein was already blocked by some remnant DNA (Fig. 3B). The highly active transcription of the *arc*_ACME_ operon, its co-transcription with the *arcR*_*ACME*_ gene, and the ability of rArcR_ACME_ to bind to the promoter region of the *arc*_ACME_ operon support the role of this protein in the activation of this important operon in the *S. aureus* USA300 clone. Moreover, the ArcR_ACME_ protein belongs to the CRP family, known for its ability to activate RNA polymerase and to facilitate the transcription process of some genes under its control (Blake et al. 2002, Akyol & Çömlekçioğlu 2009, Shimada et al. 2011).

**Fig. 3:**
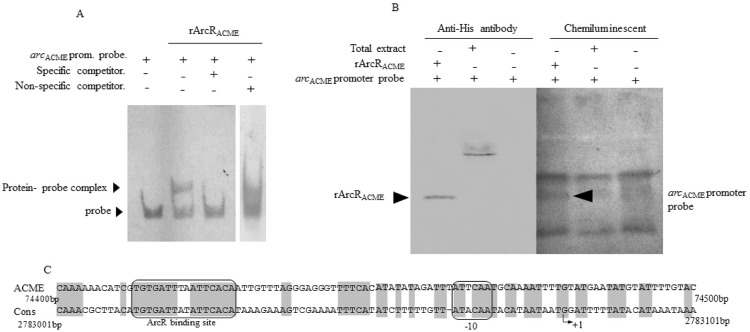
: argenine transcriptional regulator gene (*arcR*) binding to the regulatory region of arginine catabolism mobile element (*arc*ACME). (A) An electrophoretic mobility shift assay (EMSA) to evaluate mobility of the *arc*ACME promoter probe (61.2 pmol) after interaction with the rArcRACME protein (0.75 μg); the assay included a specific (unbiotinylated probe) and a non-specific competitor (*gyrB* fragment). (B) UV cross-linking of rArcRACME (0.75 µg) and a USA300 total protein extract (60 μg) with the *arc*ACME promoter region-based biotinylated probe (61.2 pmol). The interaction was visualised by non-denaturing PAGE to assess formation of the protein-probe complex. The non-denaturing PAGE was analysed by western blotting to detect the recombinant protein (with the monoclonal anti-6x-His tag antibody) and by a chemiluminescence assay (with peroxidase streptavidin and luminol) to detect the biotinylated DNA probe. (C) Partial alignment of promoter sequences of the *arc*ACME and *arc*cons operons in clone USA300. Recognition elements are surrounded by black boxes; the transcription start site is indicated as +1.

Currently, the influence of ADI on the pathogenicity of the USA300 clone (in the context of colonisation and infection) is unclear. Nevertheless, several studies suggest that the pathogenicity of this clone is mediated by its increased virulence (up-regulation of some virulence genes), which is best viewed as an adaptation to the hostile environment of the host and host’s antibacterial defences. For this reason, changes in concentration of oxygen in the medium can control virulence factor expression and the capacity for colonisation in hostile environments (Malachowa & DeLeo 2010, Lindgren et al. 2014). The reorganisation of the *arc*_ACME_ operon and the inclusion of regulator ArcR_ACME_ possibly allow for faster use of arginine and a better response to adverse environmental conditions (e.g., acidity, polyamines) through the self-activation related to a positive feedback model. This mechanism can be a contributing factor of the successful adaptation of pandemic clone USA300.
