# Supplementary material for: Participation of the arcR ACME protein in self-activation of the arc operon located in the arginine catabolism mobile element in pandemic clone USA300
Source: Mem Inst Oswaldo Cruz. 2017 Jul;112(7):499–503. doi: 10.1590/0074-02760160424 (PMC5452487; doi:10.1590/0074-02760160424)
Supplement: Supplementary file 1 [file 0074-0276-mioc-112-7-0499-suppl01.pdf]

METHODS

Assessment of relative transcription of $arcR_{ACME}$, $arcC_{ACME}$, $arcC_{cons}$ *and* $arcR_{cons}$ *genes in anaerobiosis* - Relative expression quantification of constitutive *arcR* and *arcC* genes ($arcC_{cons}$ and $arcR_{cons}$) and from ACME ($arcR_{ACME}$, $arcC_{ACME}$) was determined by quantitative polymerase chain reaction (qPCR), compared to the treated (50 mM arginine) and untreated (without arginine) *Staphylococcus aureus* USA300_FPR3757 and NCTC8325 (as control of basal expression to the constitutive genes) strains in tryptic soy broth (TSB) cultures. Relative amounts were calculated according to the method proposed by Schefe et al. (2006), normalising with the housekeeping *gyrB* gene (Fig. 1C). In detail, a single colony from USA300_FPR3757 or NCTC8325 was grown overnight in 5 mL of TSB at 37ºC, to an OD_{600} of 0.8. From these overnight cultures, 1:200 TSB dilutions (with and without arginine 50 mM) were incubated for 20 h at 37ºC under anaerobic conditions (in the case of the TSB supplemented with arginine, the pH was adjusted to 7.2 with HCl 0.5 M). The anaerobic environment was generated using the GENbag microaer system (BioMérieux®) and Resazurin (Oxoid®) was used as an indicator to verify the anaerobic condition throughout the experiment. These cultures were centrifuged at 1500 g for 7 min, the supernatant was discarded and the pellet was treated with lysozyme (10 µg/µL) for 45 min at 37ºC for bacterial lysis. RNA was extracted using the TRIzol® reagent (Invitrogen) in a final volume of 50 µL following manufacturer instructions. Prior to all experiments RNA was treated with DNAase (Promega) and the presence of genomic DNA contamination was ruled out since no *gyrB* gene amplification was observed by PCR using the RNA as template. The RNA (DNA-free) integrity was assessed through agarose gel electrophoresis. The RNA quantification was performed by absorbance at 260 nm (100-150 ng/µL) and aliquots were stored at -80ºC until use. From these RNA samples, cDNA was synthesised using reverse transcriptase (MMLV RT, 200 U/µL), a 1:4 ratio of random hexamers to RNA, 0.5 mM dNTPs, 1X buffer (50 mM Tris-HCl pH 8.3, 75 mM KCl, 3 mM $MgCl_2$ and 10 mM DTT) to a final volume of 25 µL adjusted with DEPC-treated water. This reaction was incubated for 1 h at 37ºC.

In order to perform qPCR experiments, specific primers were designed and synthetised for each gene (Supplementary data, Table II). Duplicate assays were performed for each sample (USA300_FPR3757 and NCTC8325 strains) with and without arginine using Platinum SYBR® green qPCR SuperMix-UDG (Invitrogen) on a thermocycler iQ5 (BioRad) as follows: one cycle at 95ºC (3 min), followed by 40 cycles of denaturation at 95ºC for 20 s, annealing at 57ºC for 30 s and extension at 70ºC for 30 s. Finally, melt curve analyses were performed by one cycle ramping from 65ºC to 95ºC.

Relative gene expression quantification was determined by the change in the expression of transcripts in relative units defined as "number of times" between the bacterial cultures supplemented with arginine in relation to the cultures without arginine. Relative expression was determined using the LinRegPCR Version 11.0 program, considering individual amplification efficiencies for every qPCR.

Expression, purification and detection of $rArcR_{ACME}$ *protein* - The complete sequence of the $arcR_{ACME}$ gene (without stop codon) was amplified from the USA300_FPR3757 clone (NC_007793.1) using specific primers, which contained recognition sequences for *Xba*I and *Nsi*I restriction enzymes. The 714 bp PCR amplification product was purified through the QIAkit Gel Extraction (Qiagen) following manufacturer recommendations. The $arcR_{ACME}$ gene was first cloned in pGEM®-T vector and then transformed and propagated into *Escherichia coli* TOP10 cells. The recombinant colonies (TOP10-pGEM-T-$arcR_{ACME}$) were confirmed by PCR using specific primers (Supplementary data, Table II). The pGEM-T-$arcR_{ACME}$ plasmid and pET303/CT-His vector were subjected to double digestion with *Nse*I and *Xba*I enzymes. The purified $arcR_{ACME}$ gene was then ligated to the pET303/CT-His vector according to manufacturer recommendations. The recombinant pET-CT-$arcR_{ACME}$ plasmid was transformed into both *E. coli* TOP10 and BL21 cells. A single pET-CT-$arcR_{ACME}$-BL21 colony was grown in LB broth supplemented with ampicillin (100 µg/mL) and the $rArcR_{ACME}$ (with a His_6 tag) protein expression was performed using 0.5 mM of IPTG at 37ºC at different time points (Fig. 2A). The $rArcR_{ACME}$ protein was purified through electroelution using the SnakeSkin™ Dialysis 10K (Thermo Scientific) system with TBE 0.5X, at 100V for 2 h. The $rArcR_{ACME}$ protein production was confirmed by western blot using the monoclonal anti-His_6 tag antibody (Abcam).

Total protein extract from USA300_FPR3757 - The total protein extract from USA300_FPR3757 strain was obtained as follow: a single colony was grown in BHI broth at 37ºC with shaking at 200 rpm for 24 h. Subsequently, the bacterial culture was centrifuged at 11,200 g for 10 min, the supernatant discarded and the pellet incubated with lysostaphin (50 mg/mL) and lysozyme (1 mg/mL) at 37ºC for 1 h. Then, this extract was resuspended in 800 µL of 0.1 mM Tris HCl buffer (pH 6.8) and sonicated on ice for 2 min using pulses of 30 s with intervals of 15 s and amplitude of 30%. Finally, 10 µL of protease inhibitor solution (23 mM AEBS, 100 mM EDTA, 2 mM Bestatin, 0.3 mM Pepstatin A and 0.3 mM E-64) (Sigma) was added, the extract was centrifuged at 11,200 g for 10 min, quantified (Bradford method) and evaluated by SDS-PAGE.

REFERENCES

Diep BA, Gill SR, Chang RF, Phan TH, Chen JH, Davidson MG, et al. Complete genome sequence of USA300, an epidemic clone of community-acquired meticillin-resistant *Staphylococcus aureus*. Lancet. 2006; 367(9512): 731-9.

Gillaspy AF, Worrell V, Orvis J, Roe BA, Dyer DW, Iandolo JJ. The *Staphylococcus aureus* NCTC 8325 genome. In: Fischetti VA, Novick R, Ferreti JJ, Portnoy DA, Rood JI, orgs. Gram-positive Pathogens. Washington DC: ASM Press; 2006. p. 381-412.

Schefe JH, Lehmann KE, Buschmann IR, Unger T, Funke-Kaiser H. Quantitative real-time RT-PCR data analysis: current concepts and the novel "gene expression's CT difference" formula. J Mol Med (Berl). 2006; 84: 901-10.

TABLE I

Bacterial strains and plasmids used in this study

Strain or plasmid	Genotype or relevant characteristic(s)	Source or reference
Bacterial *Staphylococcus aureus* USA300 FPR3757	*mecA*+, ACME+, ORF+, $arcC_{ACME+,}$ $arcC_{cons}$+, *arcR*+, *gyrB*+	Diep et al. (2006)
S. aureus NCTC8325	*mecA*-, ACME-,ORF-, $arcC_{ACME-,}$ $arcC_{cons}$+, *arcR*+, *gyrB*+	Gillaspy et al. (2006)
Escherichia coli TOP10	F- *mcrA* Δ(*mrr-hsdRMS-mcrBC*) φ80lacZΔM15 *ΔlacX74 nupG recA1 araD139* Δ(ara-leu)7697 *galE15 galK16 rpsL*(Str^R) *endA1* λ^-	Invitrogen
TOP10-*arcR*	F- *mcrA* Δ *mrr-hsdRMS-mcrBC*) φ80lacZΔM15 Δ*lacX74 nupG recA1 araD139* Δ (ara-leu)7697 *galE15 galK16 rpsL*(Str^R) *endA1*λ and carrying a $arcR_{ACME}$ gene.	This study
E. coli BL21(DE3)	F^- *ompT* gal dcm lon $hsdS_B(r_B^- m_B^-)$ λ(DE3 [*lacI* lacUV5-T7 gene 1 *ind1* sam7 nin5])	Invitrogen
BL21-*arcR*	F^- *ompT* gal dcm lon $hsdS_B(r_B^- m_B^-)$ λ(DE3 [*lacI* lacUV5-T7 gene 1 *ind1* sam7 nin5]) and carrying a $arcR_{ACME}$ gene.	This study
Plasmids pGEM-T Easy	An EcoRV-linearised vector with 3′ added T bases at both ends.	Promega
pET303/CT-His	Expression Vector, with T7lac promoter, C-terminal anti- 6X-His tag and amp^R.	Invitrogen
pGEM- T-$arcR_{ACME}$	pGEM-T Easy carrying a $arcR_{ACME}$ gene.	This study
pET- CT-$arcR_{ACME}$	pET303/CT-His carrying a $arcR_{ACME}$ gene.	This study

TABLE II

Primers used for plasmid construction, polymerase chain reaction (PCR), DNA probes and quantitative PCR (qPCR)

Primer	Sequence 5'à 3´	Description	Reference
$arcR_{ACME}$-for<br>$arcR_{ACME}$-rev	atgcat**tctaga**GTTATGTATGAAGAAAAT<br>TCGTGCatgcatTTTTTTATAATCCAAT	Plasmid Construction pGEM-T Easy and pET303/CT-His with $arcR_{ACME}$ gene.<br>atgcat : *Nsi*I enzyme restriction sequence.<br>**tctaga**: *Xba*I enzyme restriction sequence.	This study
SP6	CTATAGTGTCACCTAAAT	SP6 promoter primer to pGEM-T Easy.	Promega
T7 $_{promotor}$	TAATACGACTCACTATAGGG	Forward primer to pGEM-T Easy and pET303/CT-His.	Invitrogen
T7 $_{reverse}$	TAGTTATTGCTCAGCGGTGG	Reverse primer to pET303/CT-His.	Invitrogen
GP460b<br>GP461b	GTACAAAATACATATTCATAC<br>AGCGTTTTATAATGTGAATGT	Biotinylated probe in arc_{ACME} promoter region,<br>with proteins CRP/FNR consensus sequence.	This study
GP460<br>GP461	GTACAAAATACATATTCATAC<br>AGCGTTTTATAATGTGAATGT	Probe in arc_{ACME} promoter region, with proteins CRP/FNR consensus sequence.	This study
gyrB	CAAATGATCACAGCATTTGGTACAG<br>CGGCATCAGTCATAATGACGAT	Transcriptional assessment of *gyrB* gene to qPCR and construction<br>non-specific competitor probe to electrophoretic mobility shift assay (EMSA).	This study
$arcR_{ACME}$TR	GAATCTTCTAATATTACTGGTGAC<br>GTAAAGTTAATATTTTACAATCTG	Transcriptional assessment of $arcR_{ACME}$ gene to qPCR.	This study
$arcR_{cons}$TR	TGTGTACAGCATTAACCGAT TTTACTTGTTAATGCCATGT	Transcriptional assessment of constitutive *arcR* gene to qPCR.	This study
$arcC_{cons}$	TCACCGCGCGTATTGTC TGCGATTGATTTCAGTTTCCA	Transcriptional assessment of constitutive *arcC* gene to qPCR.	This study
$arcC_{ACME}$	TTTAAATTATGCGGCGGAAC GAAAGCAGAATCATCGCTTGC	Transcriptional assessment of $arcC_{ACME}$ gene to qPCR.	This study
